# CffDNA screening for Niemann–pick disease, type C1: a case series

**DOI:** 10.3389/fmed.2024.1390693

**Published:** 2024-08-05

**Authors:** Sydney A. Lau, Romy I. Fawaz, Robert Rigobello, Shahad Bawazeer, Nouf M. Alajaji, Eissa Faqeih, Yanchun Li, Yanming Feng, Fan Xia, Christine M. Eng, Malak Abedalthagafi

**Affiliations:** ^1^Baylor Genetics, Houston, TX, United States; ^2^Department of Medical Genetics, Children's Specialized Hospital, King Fahad Medical City, Riyadh, Saudi Arabia; ^3^Department of Maternal Fetal Medicine, Women's Specialized Hospital, King Fahad Medical City, Riyadh, Saudi Arabia; ^4^Department of Molecular and Human Genetics, Baylor College of Medicine, Houston, TX, United States; ^5^Department of Pathology and Laboratory Medicine, Emory School of Medicine, Atlanta, GA, United States; ^6^King Salman Center for Disability Research, Riyadh, Saudi Arabia

**Keywords:** cffDNA screening, non-invasive prenatal testing, autosomal recessive conditions, Niemann–pick disease, monogenic disorders, prenatal diagnosis

## Abstract

Cell-free fetal DNA (cffDNA) screening is a valuable tool in clinical practice for detecting chromosomal abnormalities and autosomal dominant (AD) conditions. This study introduces a novel proof-of-concept assay designed for autosomal recessive (AR) cffDNA screening, focusing on cases involving the NPC1 gene. We aim to illustrate the significant benefits of AR cffDNA screening in managing high-risk pregnancies, specifically where biallelic pathogenic variants in NPC1 cause Niemann–Pick disease, type C1 (NPC), a disorder marked by progressive neurodegeneration. Three participants for this study were recruited and gave consent to a hospital in Saudi Arabia. These participants were either carriers of NPC or had a first- or second-degree relative affected by the disorder. No specific criteria were set for the age of the participants. All were between 15 and 18 weeks of gestation. Using amplicon-based next-generation sequencing (NGS), we analyzed the zygosity and variants in cffDNA extracted from maternal peripheral blood. After amplicon NGS, analysis was completed by a custom data analysis pipeline that included in-house-built data processing scripts and commonly used software packages. Importantly, the results were not disclosed to the patients. Our findings showed that in all three cases, AR cffDNA screening results were consistent with standard invasive diagnostic testing. This screening method offers several advantages: it provides critical information to families earlier in the pregnancy compared to invasive diagnostic tests, and it helps to alleviate parental anxiety. Moreover, this non-invasive method can determine pregnancy status in the first trimester for known familial variants. Future research may extend this approach to screen for known disease-causing variants in common AR conditions.

## 1 Introduction

Introduced to the market in 2011, cffDNA screening, also known as non-invasive prenatal testing (NIPT), has become a groundbreaking method for identifying pregnancies at increased risk for specific genetic conditions. This screening can be performed as early as 9 weeks of gestation, using cffDNA circulating in maternal blood. The process involves extraction, amplification, and massively parallel sequencing to analyze cffDNA fragments and identify fetal genetic information (1).

Currently, cffDNA screening is primarily used to detect common autosomal and sex chromosome aneuploidies. Some laboratories also screen for specific microdeletion or microduplication syndromes, such as DiGeorge syndrome (2). In 2022, the American College of Medical Genetics & Genomics (ACMG) strongly recommended cffDNA screening for all pregnant individuals over traditional maternal serum screening due to its high sensitivity and specificity for aneuploidies (3).

While invasive testing via amniocentesis or chorionic villus sampling (CVS) remains the gold standard for prenatal diagnosis, cffDNA screening provides an alternative method for managing pregnancy care. Initially, cffDNA screening focused on AD conditions, such as achondroplasia. AD cffDNA screening is indicated for patients with ultrasound findings, family history, or increased risk for *de novo* conditions, often associated with advanced paternal age (4).

Despite the American College of Obstetricians and Gynecologists (ACOG) reaffirming a 2019 statement that they do not currently have sufficient information to recommend single-gene cffDNA screening during pregnancy, this screening is available for patients interested in testing for select AD conditions (5). The available AR cffDNA screening covers a limited number of conditions and is typically used as a follow-up test after traditional carrier screening (6).

Carrier screening for AR conditions is usually performed before or during pregnancy by testing both biological reproductive partners. If both partners are carriers of the same AR condition, each pregnancy has a 25% chance of being affected. Individuals without prior carrier screening or family history may be unaware that they are carriers of one or more genetic conditions. Therefore, ACMG and ACOG recommend carrier screening based on family history, ancestry, and personal preference (7). Consanguineous couples should receive genetic counseling due to their increased risk for AR conditions. High-risk results from cffDNA testing should be followed up with prenatal diagnostic testing.

Both AD and AR cffDNA screenings use similar assays involving PCR analysis (8). However, AR cffDNA screening typically requires specific probes for familial mutations and haplotype dosage analysis combined with target capture sequencing to identify conditions such as beta-thalassemia (9). AR cffDNA screening complements carrier screening and prenatal diagnostic testing by enabling early detection of genetic conditions, allowing families to make informed decisions about their pregnancies.

This study reports a proof-of-concept assay for AR cffDNA screening using peripheral blood samples from consented patients at a hospital in Saudi Arabia undergoing invasive prenatal diagnosis. In Saudi Arabia, an estimated 60% of couples are consanguineous (10). National premarital screening programs established in 2005 aim to raise awareness about the risks of passing on genetic conditions such as hemoglobinopathies and thalassemias. However, no comprehensive genetic carrier screening program is currently available in the country (11, 12). To the best of our knowledge, we only found one study in Saudi Arabia from 2010 describing the incidence rate of NPC type C as 1/100,000 live births (13).

The following three cases, involving NPC1, demonstrate the potential benefits of AR cffDNA screening in managing high-risk pregnancies. NPC1 is an AR disorder caused by biallelic pathogenic variants in the NPC1 gene, typically presenting in infancy or childhood with symptoms such as hepatosplenomegaly, failure to thrive, hypotonia, ataxia, dysphagia, and intellectual disability. There is no cure, and most treatments focus on symptom management and supportive care. NPC carriers often have mild or no symptoms but can pass the condition on to their offspring (14).

## 2 Methods

### 2.1 Recruitment

These participants were recruited from King Fahad Medical City in Saudi Arabia and were enrolled during prenatal care for the following reasons: (1) They were at increased risk of having a pregnancy affected by NPC because both reproductive partners were found to be carriers confirmed by Sanger sequencing. (2) They had a first- or second-degree relative affected by NPC. (3) No specific criteria were set for the age of the participants. Participants were in between 15 and 18 weeks of gestations.

### 2.2 Sample collection and analysis

Maternal peripheral blood was collected in Streck Cell-Free DNA BCTs^®^. CffDNA was extracted from plasma. Amplicon NGS was used to detect known familial variants; this method uses oligonucleotide probes designed to target and amplify specific regions of interest encompassing the genomic loci of known familial variants and internal reference polymorphisms, followed by NGS. After amplicon NGS, analysis was completed by a custom data analysis pipeline, which included in-house built data processing scripts and commonly used software packages such as BWA-GATK-Mutect2 or the Illumina DRAGEN Bio-IT platform. We utilized a statistical method modified from previous studies (15) to determine the variant, zygosity status of the variants, and the fetal fraction based on the NGS data.

We chose to use NGS over Q-PCR due to its ability to provide a broader and more comprehensive analysis, allowing for the detection of a wider range of genetic variations and increasing the sensitivity and specificity of our NIPT screening. This approach aligns with our goal of improving early detection and expanding the utility of NIPT.

The results from the cffDNA samples were not disclosed to the patients tested. The results of invasive prenatal testing were obtained by standard-of-care procedures.

## 3 Results

The three cases that ran under AR cffDNA screening involved *NPC1*; all were concordant between AR cffDNA screening and diagnostic testing, as shown in Table 1. Each couple conceived naturally and was expecting a singleton child, with each pregnancy being over 9 weeks gestation. We gathered thorough and complete genetic histories from each family.

**Table 1:** “Paternal status,” “Maternal status,” “cffDNA screen result,” and “Diagnostic result” refer to the presence (+) or absence (−) of the listed known familial variant (KFV) on both alleles, i.e., −/− indicates that the KFV was found on neither allele, while +/− indicates one copy of the KFV in the sample.

Case number	Gene	Variant	Paternal status	Maternal status	Fetal fraction (%)	cffDNA screen result	Diagnostic result
Case 1	NPC1	c.2130 + 1G > A	+/−	+/−	5	+/−	+/−
Case 2		c.2130 + 1G > A	+/−	+/−	7	−/−	−/−
Case 3		c.3608_3609delCA	+/−	+/−	4	+/−	+/−

*Case 1* was referred to genetics for a family history of NPC. The family history was significant for the couple’s first child, who was affected by NPC and passed away at 8 years of age. He was noted to have severe global developmental delay, dysplastic cerebral gangliocytoma, epilepsy, severe psychomotor delays, and recurrent respiratory distress; his genetic testing status was unknown. The couple was noted to be consanguineous double cousins, and neither partner had prior carrier screening. The couple’s four subsequent pregnancies underwent invasive diagnostic testing for NPC; all were found to be affected and were ultimately terminated. The sixth pregnancy resulted in an unaffected child. Case 1 was enrolled during their seventh pregnancy, and the couple pursued diagnostic testing via CVS. The CVS results confirmed the fetus to be heterozygous for *NPC1* c.2130 + 1G > A (NM_000271.4); therefore, it was expected to be a carrier for NPC. The couple was also confirmed to be heterozygous for the variant. Utilizing AR cffDNA screening, the pregnancy was predicted to be heterozygous for the *NPC1* c.2130 + 1G > A variant. The fetal fraction was 5%. To date, the female newborn has not received postnatal genetic testing and has no signs or symptoms of NPC.

*Case 2* was referred to genetics for a family history of NPC, which was significant for the pregnant partner’s nephew, who was affected by NPC and passed away at 8 years of age. The nephew was found to be homozygous for *NPC1* c.2130 + 1G > A. The couple was noted to be consanguineous first cousins. Both partners underwent carrier screening and were confirmed to be carriers of the familial *NPC1* c.2130 + 1G > A variant. Additionally, the couple has two previously unaffected children, but their carrier status is unknown. The couple opted for a CVS for this third pregnancy, and the *NPC1* variant was not detected in the fetus. The pregnancy also screened negative for the familial *NPC1* c.2130 + 1G > A variant on AR cffDNA screening. The fetal fraction was 7%. Case 2 was lost to follow-up after prenatal diagnostic testing; it is unclear whether postnatal genetic testing was done.

*Case 3* was referred for a family history of NPC; the couple’s son was affected by NPC and passed away at 4 years of age. The proband was found to be homozygous for *NPC1* c.3608_3609delCA (p.Leu1204Tyrfs*53, NM_000271.4). The proband had severe global developmental delay, persistent hepatosplenomegaly, hypotonia, lower extremity spasticity, and supranuclear ophthalmoplegia; his prenatal course was complicated by intrauterine growth restriction (IUGR). The couple did not complete carrier screening prior to pregnancy and were noted to be consanguineous first cousins. The couple also has two unaffected daughters. For this third pregnancy, the fetus was determined to be heterozygous for the familial *NPC1* variant on CVS and was expected to be a carrier for NPC. The pregnancy screened positive for a heterozygous *NPC1* c.3608_3609delCA variant on AR cffDNA screening. The fetal fraction was 4%. The couple ultimately chose to terminate the pregnancy due to other complications. It is unknown whether postmortem genetic testing was completed.

## 4 Discussion

AR cffDNA screening offers several benefits for patients. First, it provides families with critical information about their pregnancies earlier than prenatal diagnostic testing alone. This early insight is vital, as it can significantly impact decision-making processes for expectant parents. For instance, abnormal cffDNA screening results can prompt timely follow-up with diagnostic testing, either prenatally or postnatally, allowing parents to make informed decisions about their pregnancy or prepare for the required medical care after birth.

Furthermore, AR cffDNA screening can reveal the carrier status of both parents if their status for familial variants was previously unknown. This eliminates the need for separate carrier screening tests, streamlining the process and providing comprehensive genetic information in a single test. For example, in Case 1, AR cffDNA screening not only confirmed the carrier status of the parents but also clarified the familial variant, which was crucial information not available through other means.

Several studies have shown that cffDNA screening for aneuploidies has psychological and emotional benefits for patients. Women who received negative cffDNA screening results experienced decreased short-term anxiety (16). This reduction in anxiety can have a significant positive impact on the overall pregnancy experience, highlighting the psychological benefits of integrating AR cffDNA screening into prenatal care.

Despite Cases 1 and 2 sharing the same familial NPC1 variant, c.2130 + 1G > A, the two families are not related to our knowledge. The couples are from different tribes within Saudi Arabia, and this variant is neither present in ClinVar or gnomAD, nor is it listed as a founder variant on the NPC GeneReviews page. This page lists several founder variants in other populations (14). A retrospective review of children diagnosed with inborn errors of metabolism in Saudi Arabia indicated that NPC1 c.2130 + 1G > A is a founder variant in this population (17). Another study proposed that the high prevalence of AR conditions in Saudi Arabia is due to a high rate of consanguineous marriages, large family sizes, and numerous founder variants (10). These findings suggest that AR cffDNA screening may have increased utility in Saudi Arabia and culturally similar populations.

In Case 3, the fetus was heterozygous for an NPC1 variant on both AR cffDNA screening and diagnostic testing, indicating that the fetus was not affected by NPC. While the pregnancy was ultimately terminated due to other complications, the couple was able to make a more informed decision about their pregnancy options after knowing the fetus’s NPC status. This case illustrates how AR cffDNA screening can provide crucial information that aids in making difficult pregnancy decisions.

The implementation of AR cffDNA screening in clinical practice raises several ethical, legal, and social issues. The potential for this technology to provide early, non-invasive identification of AR conditions necessitates a thorough examination of its broader implications (1, 18). Consent is a crucial component of prenatal screening, particularly given the sensitive nature of genetic information and its potential impact on parental decision-making. It is essential that patients are fully informed about the benefits, limitations, and potential outcomes of AR cffDNA screening, including the possibility of false positives and the need for confirmatory diagnostic testing (19).

Ethically, the availability of such screening may influence reproductive choices and carry the risk of discrimination based on genetic information. Legal frameworks must adapt to protect patient privacy and ensure equitable access to these advanced screening methods (19). Additionally, guidelines for the clinical application of AR cffDNA screening need to be established to standardize practices and ensure consistent quality of care (20, 21).

Socially, the integration of AR cffDNA screening into routine prenatal care can have profound effects on public health policies and healthcare systems. It is important to consider the potential for increased anxiety among expectant parents and the need for appropriate genetic counseling to support them throughout the screening process (18).

With our approach to AR cffDNA screening, there are some limitations to consider. The assay was validated with a small sample size at a single center. We echo society guidelines that AR cffDNA screening is a screening test, not a substitute for prenatal or postnatal diagnostic testing, and there is not yet sufficient data to provide information regarding accuracy and predictive values in the general population (4).

CffDNA screening has been incorporated into clinical practice for chromosomal abnormalities and AD conditions. Screening for AR conditions by cffDNA is nascent in application and, to date, has not been integrated into current prenatal screening and testing care options. This method offers a non-invasive approach to discerning pregnancy status for AR conditions in the first trimester. This has the potential to benefit patients seeking information through a screening approach.

Future research should focus on continuing to optimize the concordance and predictive value of results, validating AR cffDNA screening in larger sample sizes, and conducting interviews to obtain the psychosocial impact of AR cffDNA screening in high-risk pregnancies. Additionally, as the current method is dependent on knowledge of the at-risk familial genotype, future studies may incorporate methods to screen for known disease-causing variants in common AR conditions.

## 5 Conclusion

In this study, we successfully identified fetal status for NPC1 variants during the first trimester of pregnancy using cffDNA amplicon-based testing, which matched results from routine invasive prenatal diagnosis. Our approach demonstrates the potential of this method to predict fetal risk for AR conditions. AR cffDNA screening represents a promising advancement in prenatal diagnostics, offering significant benefits for early detection and informed decision-making in high-risk pregnancies. As research and technology continue to evolve, this method has the potential to revolutionize prenatal care by providing safer, more accurate, and less invasive options for expectant families.

## Data availability statement

The raw data supporting the conclusions of this article will be made available by the authors, without undue reservation.

## Ethics statement

The studies involving humans were approved by King Fahad Medical City, Riyadh, Saudi Arabia. The studies were conducted in accordance with the local legislation and institutional requirements. The participants provided their written informed consent to participate in this study. Written informed consent was obtained from the individual(s) for the publication of any potentially identifiable images or data included in this article.

## Author contributions

SL: Writing – original draft, Writing – review & editing. RF: Writing – original draft, Writing – review & editing. RR: Writing – original draft, Writing – review & editing. SB: Investigation, Project administration, Resources, Writing – review & editing. NA: Investigation, Methodology, Resources, Writing – review & editing. EF: Investigation, Methodology, Resources, Writing – review & editing. YL: Data curation, Formal analysis, Methodology, Writing – review & editing. YF: Formal analysis, Investigation, Methodology, Writing – review & editing. FX: Conceptualization, Formal analysis, Methodology, Writing – review & editing. CE: Methodology, Writing – review & editing. MA: Conceptualization, Data curation, Formal analysis, Funding acquisition, Methodology, Project administration, Resources, Supervision, Writing – original draft, Writing – review & editing.
